# ProteoMill: efficient network-based functional analysis portal for proteomics data

**DOI:** 10.1093/bioinformatics/btab373

**Published:** 2021-05-12

**Authors:** Martin Rydén, Martin Englund, Neserin Ali

**Affiliations:** Department of Clinical Sciences Lund, Orthopedics, Clinical Epidemiology Unit, Lund University, SE-22185 Lund, Sweden; Department of Clinical Sciences Lund, Orthopedics, Clinical Epidemiology Unit, Lund University, SE-22185 Lund, Sweden; Department of Clinical Sciences Lund, Rheumatology and Molecular Skeletal Biology, Lund University, SE-22184 Lund, Sweden

## Abstract

**Summary:**

Functional analysis has become a common approach to incorporate biological knowledge into the analysis of omics data, and to explore molecular events that govern a disease state. It is though only one step in a wider analytical pipeline that typically requires use of multiple individual analysis software. There is currently a need for a well-integrated omics analysis tool that performs all the steps. The ProteoMill portal is developed as an R Shiny application and integrates all necessary steps from data-upload, converting identifiers, to quality control, differential expression and network-based functional analysis into a single fast, interactive easy to use workflow. Further, it maintains annotation data sources up to date, overcoming a common problem with use of outdated information and seamlessly integrates multiple R-packages for an improved user-experience. The functionality provided in this software can benefit researchers by facilitating the exploratory analysis of proteomics data.

**Availability and implementation:**

ProteoMill is available at https://proteomill.com.

## 1 Introduction

The large amounts of data generated from omics experiments have stressed the need for methods to reveal and extract critical components of dynamic biological systems in a readable manner, which connects to the specific study question. Expression data that are derived from high throughput analysis have multiple levels of biological features connected to it. In a real biological environment, the physical, genetic, regulatory and functional properties of a molecular set work together in a response to environmental stimuli. Holistically evaluating these attributes is a way to reveal the intercommunication between these properties and to provide a biological context. However, this task encompasses some impending challenges, including differences in biomolecule identification, data dimensionality reduction, biological contextualization, statistical analysis and data visualization and this differs among the various types of individual datasets.

Existing omics analysis tools are typically specialized for individual parts of the analysis workflow and differences in data format standards means the tools do not integrate well when used as part of an analysis workflow. This requires the researcher not only to have knowledge of the different individual software, but also knowing how to format the generated output from one software for use in the next software. This often poses a time-consuming task, particularly for researchers with little computational experience or little experience with the software(s) in question and is prone to errors.

Omics analysis platforms such as Perseus (Tyanova and Cox, 2018) and Qlucore (Qlucore, 2021) offer thorough analytical and explorative features, but require users to download and install their software and is not open source. While there are many existing web-based omics tools which are able to perform individual parts of an analysis workflow (Efstathiou *et al.*, 2017; Kuleshov *et al.*, 2016; Luo *et al.*, 2017; Merico *et al.*, 2010; Perlasca *et al.*, 2019; Schweppe *et al.*, 2017; Zheng and Wang, 2008), many lack the ability to perform complete pipelines in fast, interactive web-environments. Reimand *et al*. lists the protocols and time consumption for popular enrichment software, with the time expense ranging from minutes to several hours (Reimand *et al.*, 2019). In contrast, the run time for ProteoMill functions are a few seconds at the most, as described in Table 1.

**Table 1.** Benchmark results of ProteoMill functions

Function	Exec. time (ms)	Total time (ms)
Upload data	2560	––
Set missing value cutoff	90	––
Differential expression (limma)	215	––
Differential expression (DESeq2)	5042	––
Pathway over-representation	367	––
Interaction network^a^	53	1041
Interaction network^b^	58	5580

a Using 162 nodes and 582 edges.

b Using 1356 nodes and 17 718 edges.

Another important but often overlooked aspect for generating reliable and biologically relevant results is the quality of annotation data, and, by extension, a tool’s ability to maintain annotation data sources up to date. Lina Wadi *et al*. reported that 67% of publications in their survey referenced software using outdated annotation data (Wadi *et al.*, 2016). Web-based tools have an inherit advantage in that back-end data sources can be dynamically updated without requiring manual action by the user (such as downloading and installing software).

Analysis of proteomic data faces additional challenges (Kirik *et al.*, 2012). Different gene- and protein level identifier types are utilized in the various omics tools, which often require the researcher to convert between identifier types before proceeding to the next step of the analysis. This can result in loss of data since there can exist one-to-many mappings between two identifier types or that an identifier cannot be mapped between two identifier types (Reimand *et al.*, 2019). Furthermore, a frequent concern in mass spectrometry-derived data is the abundance of missing values (Lazar *et al.*, 2016; Wang *et al.*, 2017).

Thus, a tool that could help to transform the biological research into integrated framework is preferred. The aim of this study is to describe a newly developed software that addresses many of the existing shortcomings. The fundamental concepts of this software are to provide sets of well-integrated, easy-to-use and to a large extent automated functions for exploratory analysis of proteomic data.

## 2 Materials and methods

### 2.1 Architecture

ProteoMill runs as a web application using Shiny Server and is hosted on Amazon Web Services. The software is developed in R (version 3.6.1) and the interface was created using the R-package Shiny (Chang *et al.*, 2021) and shinydashboard (Chang and Ribeiro, 2018) (version 0.7.1) with a customized CSS theme. Animations were created using jQuery and the library animejs. Plotly (Plotly Technologies Inc., 2015), ggplot2 (Wickham, 2009), heatmaply (Galili *et al.*, 2018), networkD3 (Allaire *et al.*, 2017) and visNetwork (Almende et al., 2019) were used for plotting.

### 2.2 Identifier conversion

The Bioconductor packages AnnotationDbi (Pagès *et al.*, 2020) and ensembldb (Rainer *et al.*, 2019) was used for converting between identifiers. The identifier type of the user’s uploaded data is automatically recognized and converted to four different identifier types (where applicable). This way, the user can choose to display protein labels as any of the five identifier types, but do not need to worry about manually converting between identifiers.

### 2.3 Data quality control

Principal component analysis (PCA) was implemented using the R-package stats. Another package, mixOmics, was used for multilevel PCA.

### 2.4 Differential expression analysis

Two R-packages, limma (Ritchie *et al.*, 2015) and DESeq2 (Love *et al.*, 2014) were implemented for differential expression analysis. Each package is commonly used for fitting gene-wise linear models to expression data. limma was originally developed with a primary focus on the analysis of microarray data, while DESeq2 for the analysis of RNA-seq data and is based on the negative binomial distribution.

Differential expression analysis is conducted by specifying two contrasts and choosing a paired or non-paired design. The results are evaluated by inspecting the table in the ‘Differential expression’ tab.

The results are displayed as estimated by the specific software, using the software’s default settings for shrinkage parameters, correction for multiple testing, significance level and etc. For example, the correction for multiple testing is done using the Benjamini–Hochberg method and is applied to the tests performed within one run of the analysis and not with respect to all tests performed within one family of hypotheses in a study, which sometimes may be misleading (Ranstam, 2016). The user needs to verify if these setting are appropriate for the specific analysis done.

### 2.5 Functional enrichment and network analysis

The hypergeometric distribution was used to calculate the probability of protein list overlap.

(1) $$P=\frac{\left(\begin{array}{c} M\\ x \end{array}\right)\left(\begin{array}{c} N-M\\ n-x \end{array}\right)}{\left(\begin{array}{c} N\\ n \end{array}\right)}$$

In this formula, *N* is the total number of proteins in the background distribution, *M* is the number of proteins in the background distribution annotated to a pathway, *n* is the total number of selected proteins of interest and *x* are the proteins of interest annotated to a pathway.

Pathway data and interaction data are dynamically collected from Reactome (Fabregat *et al.*, 2018) and STRING (Szklarczyk *et al.*, 2015) (https://reactome.org/download-data). MD5sum hashes are used to ensure that the local database is up to date.

For each entry in the main pathway data file, the top-level parent pathway was annotated. This was done by creating a directed acyclic graph object using the R-package igraph (Csárdi and Nepusz, 2006).

### 2.6 Data sources

An important aspect of this software is to maintain data sources up to date. This is done by using an automated workflow at a bi-monthly interval. Data are collected from the two primary data sources, Reactome (Fabregat *et al.*, 2018) for pathway data and STRING (Szklarczyk *et al.*, 2015) for protein interaction data. These data are then structured to a predefined format, making it possible to integrate them in the analysis.

## 3 Results

The presented software, ProteoMill, proposes a unique approach to conducting explorative analysis of proteomics data. The data visualization capabilities present in this software are designed to make it possible even for researchers without any particular computational training to gain insights about the biological meaning of their data. Many of the graphical components are interactive, which is a useful feature for analysing protein interactions and selecting subnetworks of interest.

A common goal in many of ProteoMill’s functionalities is to reduce data complexity, and to provide a framework for extracting elements of biological relevance. PCA reduces a dataset of hundreds or thousands of expression datapoints into a single datapoint for each condition, plotted in 2–3 principal components, which in turn describes the dimensions with largest variability. The datapoints cluster together based on the similarity of their expression profiles.

Categorizing proteins into biological entities, described as pathways, is another way to reduce complexity and make sense of one’s data. Network graphs produced from interaction data can be difficult to interpret. In ProteoMill, pathways are used to categorize and label groups of interacting proteins, and as a way to inspect subnetworks based on these common biological themes.

The integrated enrichment- and network analysis provides a way for users to simultaneously explore functional analysis output and interaction data, and this feature has been specifically designed to easily identify and select subnetworks of interest for further analysis. 

3.1 Reproducibility

ProteoMill supports the use of reproducibility tokens as a simple way to load settings and database versions from a prior session. The token contains information about all user defined settings that affect the outcome of the analysis—every statistical result and its graphical representations. The token also contains an MD5sum hash for the uploaded dataset and warns the user if the uploaded file is not identical to the file used in the previous session. 

3.2 Performance

To assess the performance of ProteoMill, we measured the execution speed of its most prominent functions directly on the server (Table 1), using a publicly available dataset consisting of 12 samples and 12 320 proteins (Wertheim *et al.*, 2009). The time elapsed for rendering plots depends on the client-side machine and browser. The column labeled ‘Exec. Time’ describe the elapsed time of server-side calculations/data sub-setting operations and the column ‘Total time’ also describes the rendering time as measured on a 2018 MacBook Pro (2.2 GHz 6-Core Intel Core i7).

## 4 Discussion

The integrated features in this software provide powerful visualization strategies for the exploration of omics data, with a particular focus on the management and manipulation of proteomics data. By using this platform, researchers can expect to discover biologically relevant rendering of their data through results aggregated from reliable and up-to-date data sources.

The software offers innovative strategies to interactively explore quantitative proteomics data in a comprehensive workflow from data-upload to network analysis. It has a strong focus on well-maintained data sources, computational efficiency and user-friendliness.

Importantly, ProteoMill utilizes many existing R packages for statistical analysis and pathway annotation that are standard in the field. However, these methods are strongly focused on estimation of P-values and classifications of results based on P-value thresholds. This is an unfavorable approach to use of statistical methods and there is a need to move further in better estimation methods and expressing uncertainty (Benjamin *et al.*, 2018).
